# The hemoglobin glycation index stratifies heart failure phenotypes and in-hospital risk

**DOI:** 10.3389/fendo.2025.1740447

**Published:** 2025-12-15

**Authors:** Chuxin Lyu, Xinyu Tong, Pingyang Fu, Yuan Gao, Jiayi Hua, Jiajing Zhao, Peng Yu, Xiaohu Chen, Dongling Lyu

**Affiliations:** 1 Department of Cardiology, Affiliated Hospital of Nanjing University of Chinese Medicine, Nanjing, Jiangsu, China; 2 Department of Cardiology, Jiangsu Province Hospital of Chinese Medicine, Nanjing, Jiangsu, China; 3 First Clinical Medical College, Nanjing University of Chinese Medicine, Nanjing, Jiangsu, China; 4 Department of Neurology, Wuxi Traditional Chinese Medicine Hospital, Wuxi, Jiangsu, China

**Keywords:** hemoglobin glycation index, heart failure, ejection fraction, worsening heart failure, in-hospital outcomes

## Abstract

**Objective:**

To evaluate the association between the hemoglobin glycation index (HGI) and ejection fraction (EF) categories in hospitalized heart failure (HF) patients and to investigate the relationship between HGI and in-hospital worsening heart failure (WHF).

**Methods:**

This single-center retrospective study included 647 HF patients (261 HFrEF, 186 HFmrEF, 200 HFpEF). HGI was calculated as measured HbA1c minus predicted HbA1c (derived from fasting plasma glucose regression). Ordinal multinomial logistic regression and binomial logistic regression were used to evaluate the relationships between HGI (exposure) and EF-based phenotypes and in-hospital WHF (outcome), respectively, with stepwise adjustment for confounding factors. Dose-response relationships were assessed using restricted cubic spline (RCS) analysis.

**Results:**

HFrEF patients exhibited the highest HGIs and the highest incidence of In-hospital WHF (33.72% vs. 18.82% [HFmrEF] and 14.00% [HFpEF]; P < 0.001). After full adjustment, a higher HGI was significantly associated with the HFrEF phenotype. For every 1-unit increase in HGI, the probability of being classified into a higher EF category (HFrEF → HFmrEF/HFpEF) decreased by approximately 25% (OR = 0.746, 95% CI 0.617–0.902; P = 0.003; P for trend = 0.029). Regarding outcomes, HGI showed a stable, positive association with In-hospital WHF. After full adjustment, every 1-unit increase in HGI increased the risk of In-hospital WHF 2.16-fold (OR = 2.161, 95% CI 1.680–2.840; P<0.001). When divided into quartiles (Q1 as reference), the ORs for In-hospital WHF in Q2, Q3, and Q4 were 2.790, 3.811, and 7.322, respectively (P for trend < 0.001). RCS analysis revealed an approximately linear dose–response relationship.

**Conclusion:**

In hospitalized HF patients, a higher HGI was significantly associated with the HFrEF phenotype and an increased risk of in-hospital WHF. HGI may serve as a potential supplementary indicator for phenotype characterization and risk stratification.

## Introduction

Heart failure (HF) has become an important public health problem, with continually increasing prevalence and related disease burden. The latest data show that the prevalence of HF in Chinese adults aged 35 and above is approximately 1.3%, translating to approximately 13.7 million affected individuals, an increase of 0.4% since 2000 (1). Clinically, on the basis of the left ventricular ejection fraction (LVEF), HF is classified into HF with reduced ejection fraction (HFrEF, LVEF ≤40%), HF with mildly reduced ejection fraction (HFmrEF, LVEF 41–49%), and HF with preserved ejection fraction (HFpEF, LVEF ≥50%) (2, 3); the different phenotypes are characterized by significant heterogeneity in terms of etiology, pathophysiology, and treatment responses (4–6). This heterogeneity suggests that phenotype-related metabolic or molecular markers could help elucidate the mechanisms underlying the phenotypes and improve phenotype-based diagnosis and treatment strategies.

Myocardial and vascular remodeling involving abnormal glucose metabolism and advanced glycation end products (AGEs) is considered a key pathway in the occurrence and development of HF (7). AGEs interact with their receptors (receptors of AGEs, RAGEs) to induce oxidative stress and inflammatory responses (8), which cause collagen and elastin cross-linking at the extracellular matrix level, leading to arterial stiffness, myocardial fibrosis, and impaired systolic and diastolic function (9, 10). The extents of these changes are closely related to the structural and functional phenotype of HF.

However, the traditional glycated hemoglobin (HbA1c) level is affected by the inherent glycation characteristics of the individual, and long-term and stable, systemic differences in HbA1c levels may present among individuals with the same blood glucose levels (11). To quantify the individual differences, Hempe et al. (12) proposed the hemoglobin glycation index (HGI), defined as the difference between the measured HbA1c and the HbA1c predicted by regression analysis based on blood glucose (mean or fasting), to reflect individualized glycation sensitivity.

The HGI has also been introduced into investigations of HF as an index that could be adjusted for when addressing individual differences. A series of retrospective studies yielded preliminary evidence of an association between HGI abnormalities and adverse outcomes in HF patients. In a study including 2846 HF patients admitted to the intensive care unit (ICU), Guo et al. reported that those with a high HGI had significantly higher 30-day and 1-year mortality rates than those with a low HGI did (13). Conversely, another study based on the MIMIC-IV database suggested that an excessively low HGI also predicts poor outcomes (14). Taken together, the results of these studies indicate that the HGI can be used as a valuable supplementary indicator for evaluating the outcomes of HF patients. However, the existing literature has focused mainly on the relationship between the HGI and mortality of HF patients, and the study populations have mostly included patients with diabetes mellitus or in the ICU; there is limited evidence to support an association between the HGI and specific HF phenotypes (HFrEF, HFmrEF, HFpEF), and research on the phenotypic distribution of hospitalized HF patients, especially from Chinese populations, is limited. On this basis, we conducted a retrospective study on HF patients admitted to Jiangsu Hospital of Traditional Chinese Medicine from 2021 to 2025 to evaluate the association between the HGI and different HF phenotypes and to clarify the predictive value of the HGI for in-hospital adverse events.

## Methods

### Study population

**Figure 1:**
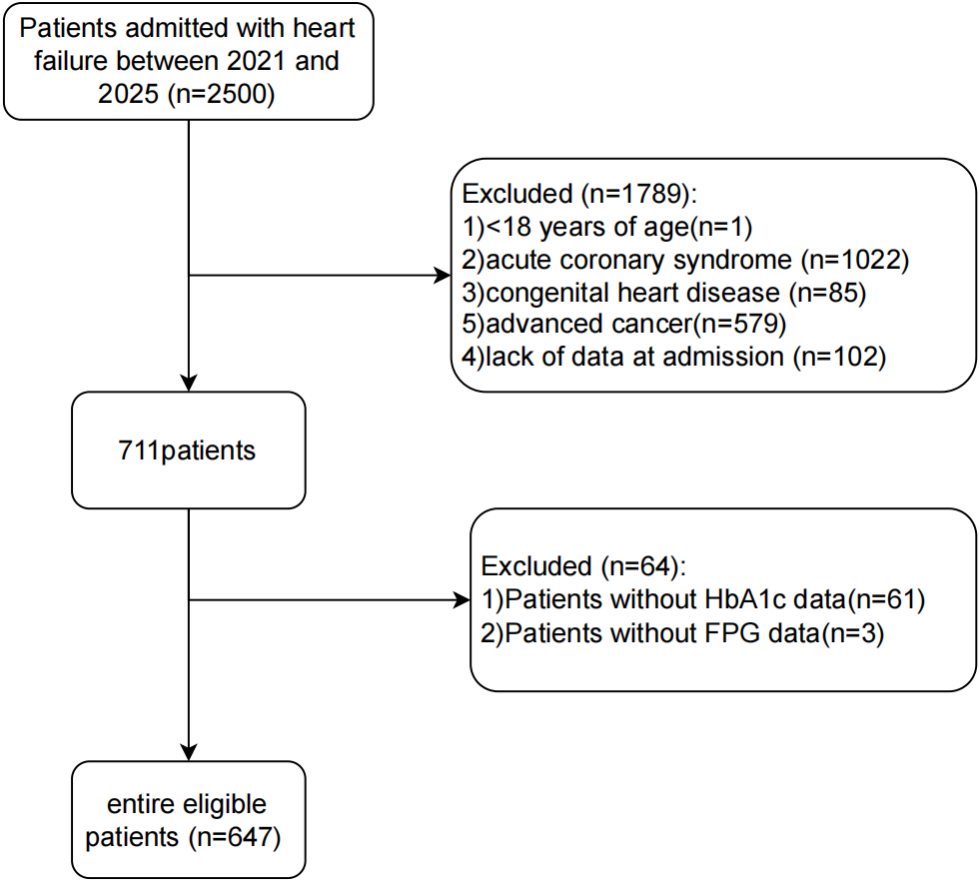
Flowchart of patient selection.

This was a single-center retrospective cohort study. HF patients hospitalized in the Department of Cardiology, Jiangsu Hospital of Traditional Chinese Medicine, from January 2021 to June 2025 were consecutively enrolled. The exclusion criteria were (1) age <18 years, (2) acute coronary syndrome, (3) congenital heart disease, (4) advanced malignancies, (5) lack of data at admission, and (6) loss to follow-up. A total of 2500 patients hospitalized for HF were enrolled in this study, of whom 1853 were excluded for different reasons, and 647 patients were finally included in the analysis (**Figure 1**).

This retrospective study followed the Declaration of Helsinki and was approved by the Ethics Committee of Jiangsu Hospital of Traditional Chinese Medicine (approval number: 2025NL-068-02). (Appendix S1).

### Data collection and definitions

Baseline data, including demographic information, medical history, laboratory test data, and echocardiography data, were extracted from the hospital’s electronic health care record system by two experienced doctors. Demographic information included age, sex, height, and weight. Past medical history, including diabetes mellitus, hypertension (HTN), coronary heart disease, hyperlipidemia, and chronic kidney disease, was obtained through standardized questionnaires.

The laboratory indexes included the levels of HbA1c, fasting plasma glucose (FPG), triglyceride (TG), total cholesterol (TC), high-density lipoprotein cholesterol (HDL-C), low-density lipoprotein cholesterol (LDL-C), N-terminal proB-type natriuretic peptide (NT-proBNP), D-dimer (DD), urea nitrogen (BUN), aspartate aminotransferase (AST), albumin (ALB), total bilirubin (TBIL), creatine kinase isoenzyme (CK-MB), and high-sensitivity C-reactive protein (hs-CRP), white blood cell (WBC) count, red blood cell (RBC) count, hemoglobin (Hb) level, platelet count (PLT), absolute lymphocyte count (Lym), absolute neutrophil count (NEU), and absolute monocyte count (MO). All these indexes were measured using the peripheral venous blood samples collected from the first blood draw after admission. Echocardiography was used to measure the LVEF.

### HGI calculation

**Figure 2:**
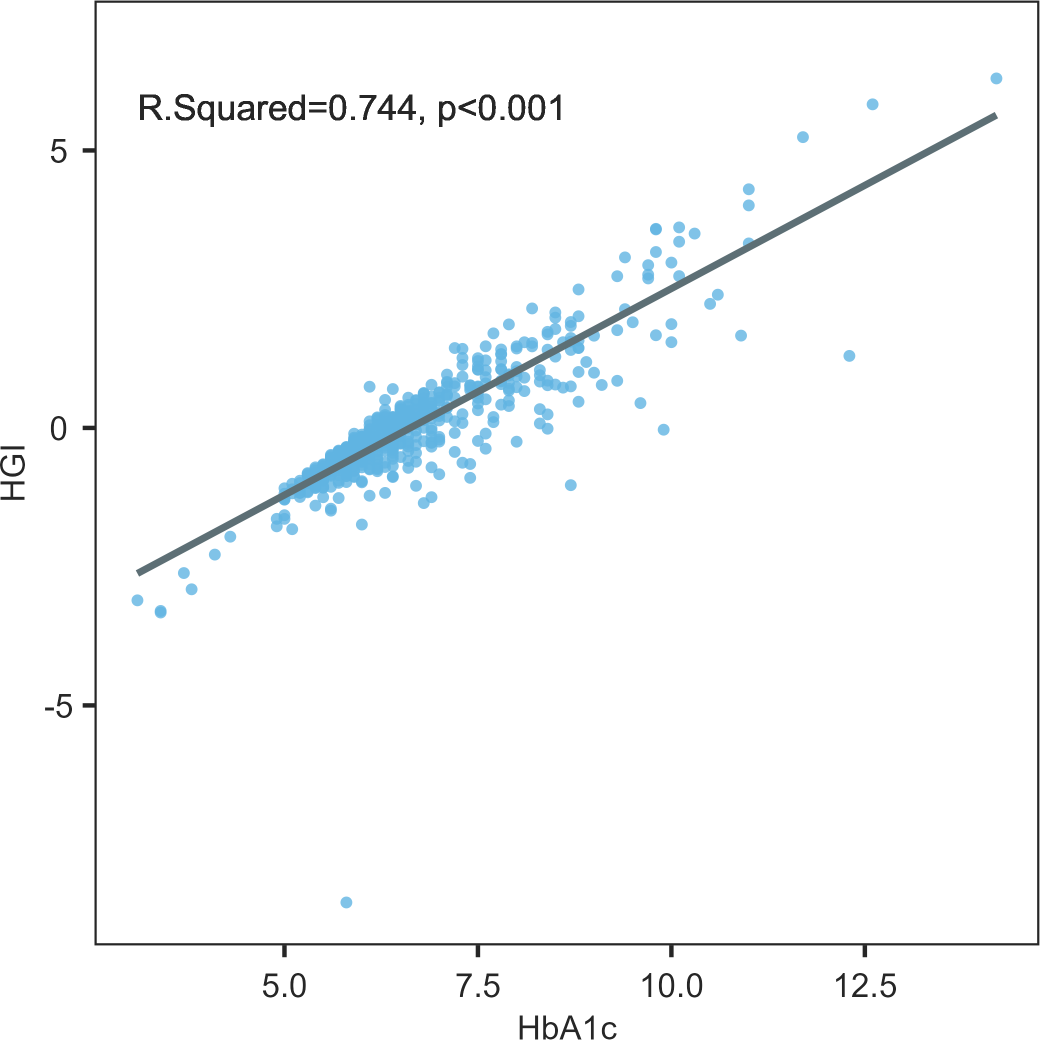
Relationship between HGI and HbA1c in the study cohort.

The HGI was calculated using the method proposed by Hempe et al. (12). The linear relationship between the baseline FPG and HbA1c data of all the subjects were assessed in the population of this study. The predicted HbA1c was calculated using the following formula: predicted HbA1c = 0.2046 × FPG + 5.3290. The difference between the observed and predicted HbA1c levels was subsequently defined as the HGI. The correlation between the HGI and the level of HbA1c is shown in **Figure 2**.

### Variables (exposures and covariates)

The primary exposure variable was the HGI. HF was classified into HFrEF (<40%), HFmrEF (40–49%), and HFpEF (≥50%) according to the LVEF measured by the most recent ultrasound within 72 hours after admission. The results, which served as the baseline phenotype variables, were included in the description, stratification, and interaction analyses.

### Outcome definitions

During hospitalization, in-hospital worsening heart failure were defined as the occurrence of any of the following composite endpoints during this indicated hospitalization: (1) additional use of intravenous diuretics during the emergency department visit or hospitalization; and 2) the need for positive inotropes (dobutamine/milrinone, etc.) or mechanical circulatory support.

### Statistical analysis

First, the baseline data were compared between groups. Missing data were handled using complete-case analysis. Patients with missing values for the primary exposure (HbA1c, FPG) or outcome variables were excluded during the cohort selection process. In the final analytic sample (n=647), the rate of missing data for all included covariates was 0%. Continuous variables were tested for normality (Shapiro–Wilk test) and homogeneity of variance (Levene’s test). Normally and approximately normally distributed variables are expressed as the mean ± standard deviation and were analyzed by one-way analysis of variance (ANOVA), followed by Tukey’s honestly significant difference (HSD) test for *post hoc* pairwise comparisons. Variables with skewed distributions are expressed as medians (quartiles) and were analyzed by the Kruskal–Wallis test. Categorical variables are expressed as frequencies (%) and were analyzed with the χ² test or Fisher’s exact test.

The primary exposure, HGI, was analyzed as both a continuous variable and categorized into quartiles (Q1–Q4, with Q1 as the reference). The primary outcomes investigated were the association with EF-based HF phenotypes and the risk of in-hospital WHF.

To evaluate the association between HGI and HF phenotypes, ordinal multinomial logistic regression was used. Each phenotype was assigned an ordinal number that served as the dependent variable (HFrEF = 1, HFmrEF = 2, HFpEF = 3).

To investigate the relationship between HGI and in-hospital outcomes, binary logistic regression was performed with the occurrence of In-hospital WHF as the outcome. Results from both regression analyses are reported as the odds ratios (OR) and 95% confidence intervals.

For both modeling approaches, three-step adjustments were performed according to the preset stratification, yielding three models: Model 1 was the unadjusted model; Model 2 was derived from Model 1 by adjusting for age, sex, HTN, type 2 diabetes mellitus (T2DM), ischemic heart disease (IHD), hyperlipidemia (HLD), and chronic kidney disease (CKD); and Model 3 was obtained by further adjusting Model 2 for New York Heart Association (NYHA) classes and TC, HDL-C, LDL-C, BUN, Cr, AST, ALB, CK-MB, NT-proBNP, hs-CRP, RBC, and Hb levels. When analyzing HGI by quartiles, the linear comparison method was used for trend analysis (P for trend), in which the median within each quantile group was included as a continuous variable in the regression calculation.

To assess the potential nonlinearity and dose–response relationship between the HGI and the outcomes, restricted cubic spline (RCS) analysis was used to model HGI as a spline function. The exposure–response curves and the 95% confidence intervals were plotted for the above three models (Models 1–3 of multinomial and binary logistic regression). The overall effect and the nonlinear component were obtained through the likelihood ratio test (the overall P value and the nonlinear P value are reported separately), and the reference value was set to be the predetermined reference HGI.

All tests were two-sided (α = 0.05). All the data were analyzed using R 4.5.1 and SPSS for Windows 27.0. P < 0.05 was considered to indicate statistical significance.

## Results

### Baseline characteristics of the study cohort

A total of 647 patients with HF were included in this study, including 261 HFrEF patients, 186 HFmrEF patients, and 200 HFpEF patients. The three groups of patients showed significant differences in many clinical and laboratory indicators.

In terms of general clinical characteristics (**Table 1**), HFpEF patients were the oldest (79 years old), followed by HFmrEF patients (76 years old), and HFrEF patients were the youngest (71 years old; p <0.01). The proportion of males was the highest in the HFrEF group (73.18%) and the lowest in the HFpEF group (54.00%, p <0.01). The LVEF tended to gradually increase across the ordinal EF categories (HFrEF 31%, HFmrEF 45%, and HFpEF 54%, p <0.01). The differences in body weight and height among the groups were also statistically significant. In terms of laboratory indicators, HFrEF patients had higher HbA1c levels (6.4% vs. 6.2%, p < 0.01), while HFpEF patients had the lowest Hb level (114 g/L, p < 0.01). The RBC also decreased across the different phenotypes (p=0.02). The differences in LDL, FPG, BUN, Cr, and UA levels and inflammatory indicators (hs-CRP level, WBC count, NEU, Lym, and MO) among the three groups were not statistically significant. The level of NT-proBNP was the highest in the HFrEF group (7210 vs. 2690 pg/mL, p <0.01). The HGI was greatest in the HFrEF group and lower in the HFmrEF and HFpEF groups (p < 0.01). In terms of comorbidities, HTN was the most prevalent in the HFpEF group (85.5%, p <0.01), while T2DM was the most prevalent in the HFmrEF group (46.77%, p = 0.01). IHD was most common in the HFmrEF group (59.68%, p=0.04), and CKD was most common in the HFrEF and HFpEF groups (approximately 40%, p=0.04). The incidence of In-hospital WHF was highest in HFrEF patients (33.72%, p < 0.01).

**Table 1:** Patient baseline information.

Variable	Level	Overall	HFrEF	HFmrEF	HFpEF	P
n		647	261	186	200	
Age, years		76 (66-82)	71 (62-78)	76 (67.5-83)	79 (72-84)	<0.001
Male, n (%)	Yes	407 (62.91)	191 (73.18)	108 (58.06)	108 (54.00)	<0.001
EF (%)		43 (34-51)	31 (26-35)	45 (42-47)	54 (52-58)	<0.001
Weight, kg		66 (60-75)	67 (60-77)	65 (60-73.75)	66 (58.75-74.62)	0.025
Height, cm		166 (160-172)	168 (160-173)	165 (160-170)	165 (160-170)	<0.001
TG, mmol/L		0.99 (0.75-1.35)	1 (0.79-1.35)	1.02 (0.73-1.4)	0.96 (0.7-1.31)	0.331
TC, mmol/L		3.45 (2.88-4.22)	3.6 (3.01-4.33)	3.42 (2.82-4.21)	3.35 (2.84-3.97)	0.289
HDL, mmol/L		1.08 (0.88-1.31)	1.05 (0.88-1.26)	1.1 (0.89-1.33)	1.1 (0.86-1.34)	0.695
LDL, mmol/L		1.85 (1.46-2.42)	2.03 (1.61-2.57)	1.81 (1.41-2.37)	1.67 (1.34-2.15)	0.275
FPG, mmol/L		5.49 (4.68-6.94)	5.59 (4.73-7.07)	5.53 (4.74-7.18)	5.33 (4.53-6.55)	0.252
HbA1c		6.3 (5.9-7)	6.4 (5.9-7.5)	6.3 (5.9-7)	6.2 (5.8-6.62)	<0.001
D_Dimer,mg/L FEU		0.94 (0.4-1.81)	1.03 (0.46-1.86)	0.9 (0.36-1.42)	0.92 (0.37-1.88)	0.191
BUN, mmol/L		8.58 (6.46-12.72)	8.81 (6.77-12.87)	8.03 (6.06-12.14)	8.58 (6.43-12.99)	0.160
Cr, μmol/L		101.2 (80.45-145.9)	99.7 (82.6-141.6)	98.3 (79.73-148.1)	106.05 (80.58-151.02)	0.533
UA, μmol/L		424 (340-536)	448 (368-576)	388 (311.25-492.75)	414.5 (338-506.25)	0.191
ALT, U/L		18 (12-31)	22 (14-39)	17 (12-26)	17 (10.75-24.25)	0.406
AST, U/L		23 (18-31.5)	25 (19-36)	22 (18-29)	22 (17-28.25)	0.488
ALB, g/L		37.4 (34.3-40.15)	38 (34.4-40.2)	36.85 (33.92-40.08)	37.3 (34.48-39.9)	0.588
Tbil, μmol/L		14.15 (9.64-21.31)	15.87 (11-24.21)	13.04 (9.3-19.25)	12.86 (8.51-19.29)	0.049
CK_MB, U/L		13 (10-18)	14 (11-19)	13 (9.05-17)	13 (10-16)	0.229
hs_CRP, mg/L		1.76 (0.5-8.96)	1.98 (0.5-8.31)	1.73 (0.5-10.75)	1.54 (0.5-8.17)	0.126
WBC,×10^9^/L		6.05 (4.88-7.7)	6.13 (5.14-7.65)	6.12 (4.82-7.99)	5.85 (4.7-7.55)	0.178
RBC,×10^9^/L		4.13 (3.54-4.74)	4.38 (3.95-4.95)	4.1 (3.55-4.6)	3.73 (3.14-4.41)	0.016
Hb, g/L		126 (105-143)	135 (119-149)	125 (105-142)	114 (91.75-133)	0.001
PLT,×10^9^/L		170 (136-208.5)	170 (140-205)	167 (129.5-207)	175 (140-216.5)	0.652
Lym,×10^9^/L		1.14 (0.81-1.6)	1.26 (0.86-1.66)	1.12 (0.78-1.6)	1.04 (0.77-1.49)	0.328
NEU,×10^9^/L		4.05 (3.2-5.43)	4.04 (3.34-5.3)	4.11 (2.93-5.6)	3.96 (3.1-5.31)	0.317
MO,×10^9^/L		0.49 (0.37-0.65)	0.51 (0.39-0.65)	0.48 (0.35-0.69)	0.47 (0.36-0.61)	0.795
NYHA		3 (3-3)	3 (3-4)	3 (3-3)	3 (3-3)	<0.001
NT_pro_BNP, pg/mL		4690 (1755-10300)	7210 (3390-15960)	3729 (1670-8642.5)	2690 (1245-6359)	<0.001
HGI		-0.17 (-0.57-0.35)	-0.08 (-0.53-0.67)	-0.24 (-0.65-0.35)	-0.22 (-0.58-0.15)	<0.001
HTN (%)	1	485 (74.96)	167 (63.98)	147 (79.03)	171 (85.50)	<0.001
T2DM (%)	1	262 (40.49)	112 (42.91)	87 (46.77)	63 (31.50)	0.006
IHD (%)	1	347 (53.63)	142 (54.41)	111 (59.68)	94 (47.00)	0.042
HLD (%)	1	94 (14.53)	36 (13.79)	34 (18.28)	24 (12.00)	0.197
CKD (%)	1	241 (37.25)	106 (40.61)	55 (29.57)	80 (40.00)	0.037
In-hospital WHF (%)	1	151 (23.34)	88 (33.72)	35 (18.82)	28 (14.00)	<0.001

### Relationship between the HGI and LVEF

HGI by EF-Stratified HF: One-Way ANOVA with Tukey’s HSD

**Figure 3:**
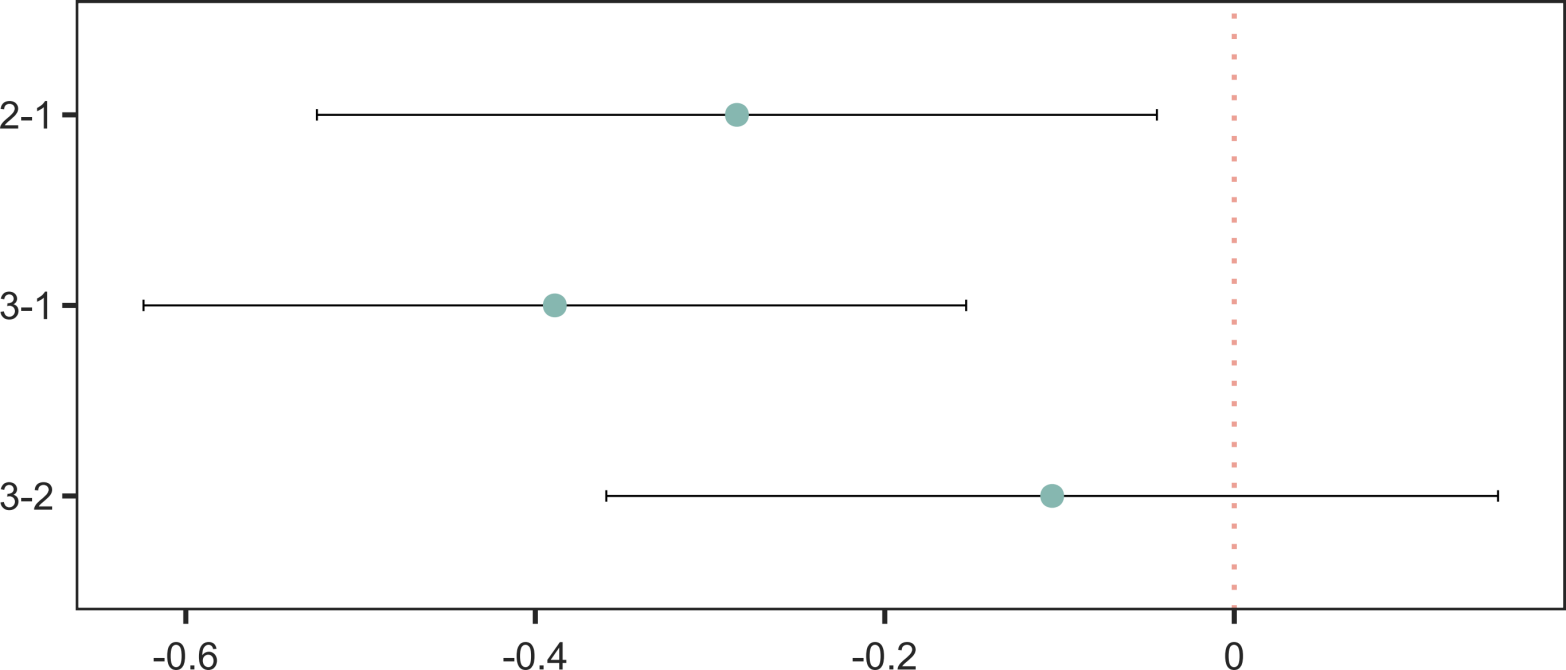
Pairwise differences in HGI across heart-failure phenotypes.

The HGI was used as the dependent variable to assess differences among different phenotypes of HF. One-way ANOVA revealed significant differences in the mean HGI values among the three groups (F(2,644)=8.3115, MSE = 1.1370, partial η²=0.0252, P = 0.0003), indicating that overall, the HGI differed among the HF phenotype groups. Tukey’s HSD was further used for pairwise comparisons (**Figure 3**): compared with eflevel 1, the mean difference in the HGI of eflevel 2 was -0.2846 (95% CI: -0.5250~ -0.0443; P_adj = 0.0153), and the mean difference in the HGI of eflevel 3 was -0.3888 (95% CI: -0.6242 to -0.1534; P_adj = 0.0003), whereas the difference between eflevel 3 and eflevel 2 was not significant (the mean difference was -0.1042; 95% CI: -0.3593 to 0.1510; P_adj = 0.6029). The above results suggest that compared with eflevel 1 patients, eflevel 2 and eflevel 3 patients had lower HGIs, while there was no significant difference in the HGI between eflevel 2 and eflevel 3 patients.

### Multinomial logistic regression

In the multinomial logistic regression of the data of the included HF patients (HFrEF → HFmrEF → HFpEF), the HGI, when treated as a continuous variable, was independently and negatively correlated with the probability of a patient transitioning to a higher EF category; after adjustment for all confounding factors, for every 1 unit increase in the HGI, the probability of being classified into a higher EF category was reduced by approximately 25% (OR = 0.746, 95% CI 0.617–0.902; P = 0.003). Grouping the HGI according to quartiles, a significant linear trend emerged (fully adjusted model: P for trend = 0.029), where only the odds of progressing to a higher EF category was significantly negatively associated with the highest quartile (Q4) (OR = 0.470, 95% CI 0.281–0.786, P = 0.004), and there was no statistically significant difference for Q2 and Q3. The above direction and significance were generally consistent in models with different levels of adjustment (see **Table 2**).

**Table 2:** Association between HGI and EF-stratified HF phenotype.

Variables	Model 1		Model 2		Model 3	
	OR (95% CI)	P value	OR (95% CI)	P value	OR (95% CI)	P value
HGI continuous	0.757(0.656, 0.874)	<0.001	0.797(0.675, 0.941)	0.007	0.746 (0.617, 0.902)	0.003
HGI group	P for trend: 0.004		P for trend: 0.036		P for trend: 0.029	
Q1						
Q2	0.983 (0.659,1.468)	0.935	0.924 (0.609,1.403)	0.711	0.831 (0.534,1.292)	0.411
Q3	1.147 (0.767,1.716)	0.505	1.122 (0.738,1.706)	0.589	1.042 (0.664,1.634)	0.859
Q4	0.507 (0.339,0.759)	0.01	0.517 (0.325,0.823)	0.006	0.470 (0.281,0.786)	0.004

Model 1: unadjusted.

Model 2: adjusted for age, sex, HTN, T2DM, IHD, HLD, and CKD.

Model 3: adjusted for age, sex, HTN, T2DM, IHD, HLD, CKD, NYHA, TC, HDL-C, LDL-C, BUN, Cr, AST, ALB, CK-MB, NT_pro_BNP, hs-CRP, RBC and hb.

**Figure 4:**
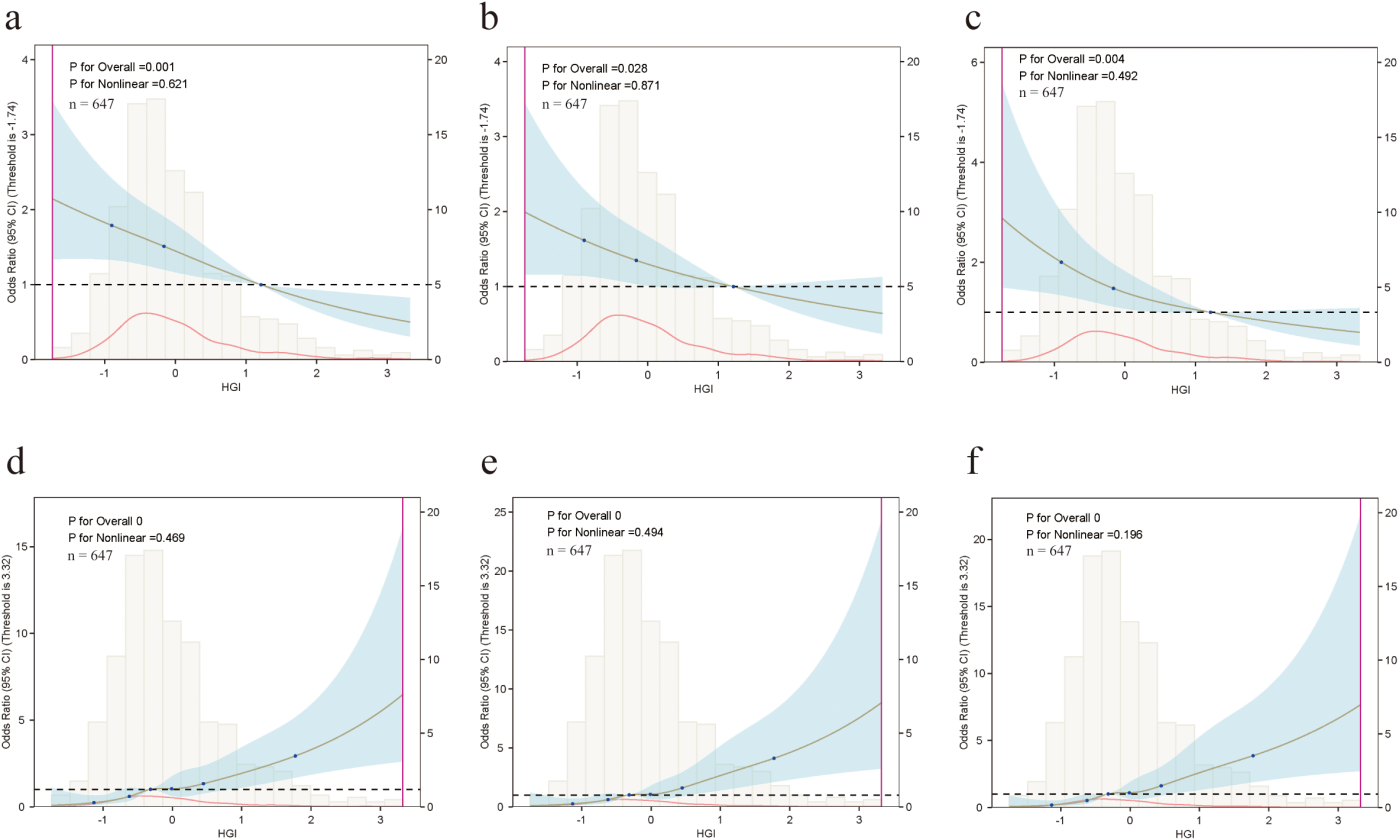
RCS analysis of the association between HGI and HF phenotype and in-hospital WHF (Models 1–3). **(a–c)** Association between HGI and HF phenotypes in Models 1, 2, and 3, respectively. **(d–f)** Association between HGI and in-hospital WHF in Models 1, 2, and 3, respectively.

In the ordinal multinomial logistic model of HF classification (a, b, and c correspond to Models 1–3, respectively), the HGI and phenotype number (1 → 3) demonstrated a monotonically decreasing and nearly linear relationship; the P values of the overall correlation test were 0.001, 0.028, and 0.004; and the P values of the nonlinear test were 0.621, 0.871, and 0.492, respectively, indicating that the relationships were not nonlinear. The curve intersects with OR = 1 at a HGI value of approximately 1, and the OR remains below 1 as the HGI increases, suggesting that a high HGI is associated with a decrease in the phenotype number and that the directions are consistent across all the models. The RCS results were consistent with the main analysis results: an increase in HGI was associated with a decrease in the HF phenotype number (see **Figure 4**).

### Relationships between the HGI and the endpoints

In the retrospective analysis of hospitalized HF patients, logistic regression was performed with the occurrence of In-hospital WHF as the outcome. The results revealed that when treated as a continuous variable, the HGI was significantly positively correlated with the occurrence of In-hospital WHF (unadjusted model: OR = 1.919; 95% CI 1.589–2.347; after adjustment for age, sex, and common comorbidities: OR = 2.131; 95% CI 1.690–2.735; P < 0.001; after adjustment for all confounding variables: OR = 2.161; 95% CI 1.680–2.840; P < 0.001). The trend test with the HGI grouped in quartiles was significant in all the models (all P values for trend < 0.001). In the fully adjusted model, with Q1 as the reference, the OR values of the occurrence of In-hospital WHF in patients whose HGIs were in Q2, Q3, and Q4 were 2.790 (95% CI 1.391–5.862; P = 0.005), 3.811 (95% CI 1.902–8.034; P<0.001), and 7.322 (95% CI 3.457–16.371; P<0.001), respectively, indicating a clear dose–response relationship. After full factor adjustment, the results were consistent in direction and similar in strength, suggesting that a high HGI level was significantly and robustly associated with a greater risk of In-hospital WHF (see **Table 3**).

**Table 3:** Association between HGI and in-hospital WHF: binary logistic regression.

Variables	Model 1		Model 2		Model 3	
	OR (95% CI)	P value	OR (95% CI)	P value	OR (95% CI)	P value
HGI continuous	1.919 (1.589, 2.347)	<0.001	2.131 (1.690, 2.735)	<0.001	2.161 (1.680, 2.840)	<0.001
HGI group	P for trend: <0.001		P for trend: <0.001		P for trend: <0.001	
Q1						
Q2	2.415 (1.271,4.775)	0.009	2.298 (1.198,4.584)	0.015	2.790 (1.391,5.862)	0.005
Q3	2.904 (1.550,5.689)	0.001	2.859 (1.511,5.649)	0.002	3.811 (1.902,8.034)	<0.001
Q4	5.469 (3.013,10.483)	<0.001	5.957 (3.066,12.161)	<0.001	7.322 (3.457,16.371)	<0.001

Model 1: unadjusted.

Model 2: adjusted for age, sex, HTN, T2DM, IHD, HLD, and CKD.

Model 3: adjusted for age, sex, HTN, T2DM, IHD, HLD, CKD, NYHA, TC, HDL-C, LDL-C, BUN, Cr, AST, ALB, CK-MB, NT_pro_BNP, hs-CRP, RBC and Hb.

In the three logistic models (d, e, f) that used In-hospital WHF as the outcome, the relationship between the HGI and the occurrence of In-hospital WHF monotonically increased and was nearly linear; the P values of the overall correlation test were all <0.001 (shown as 0 in the figure); and the P values of nonlinear test were 0.469, 0.494, and 0.196, respectively. The curves were close to OR = 1 in the low-HGI range and gradually increased from approximately 0–1; this increase was most obvious more obvious in the high-HGI range until HGI≈3, when the OR was approximately >6–8. The uncertainty in the right side of the plot is high (that is, the confidence interval is relatively wide), likely due to the small sample size. An increase in the HGI was associated with an increase in the risk of developing In-hospital WHF. Finally, there was no significant nonlinear inflection point in the relationships of the HGI with either HF phenotype or the occurrence of In-hospital WHF, indicating stable dose–response characteristics (**Figure 4**).

## Discussion

This study was the first to confirm the presence of significant differences in the HGI across different HF phenotypes: the HGI of HFrEF patients was significantly greater than that of the HFmrEF and HFpEF patients, whereas the difference between the HFmrEF and HFpEF patients was not statistically significant. Multinomial logistic regression further revealed that an elevated HGI was independently and negatively associated with a greater probability of a patient transitioning to a higher EF category (HFrEF to HFmrEF/HFpEF). After full adjustment for confounding factors, the probability of being classified into a higher EF category decreased by approximately 25% (OR≈0.75). This result indicates that the higher the HGI is, the more likely it is that the patient will have the HFrEF phenotype. Additionally, we found that the HGI was significantly positively correlated with the risk of developing In-hospital WHF during hospitalization: when the HGI was used as a continuous variable, the risk of developing In-hospital WHF increased by a factor of approximately 2 with each unit increase in the HGI. It is worth noting that while the numerical distribution of HGI appears narrow (centered near zero), a 1-unit increase in HGI represents a 1% absolute difference between observed and predicted HbA1c. Biologically, this reflects a substantial deviation in individual glycation susceptibility. Given this distribution, our use of quartile analysis confirms that even smaller increments in HGI (moving from Q1 to Q4) are associated with a steep gradient in risk, reinforcing the clinical relevance of HGI variations within the sub-unit range. In summary, a high HGI may suggest that the patient is more likely to have the HFrEF phenotype and have a higher risk of experiencing short-term adverse events, whereas patients with low HGIs are more likely to have the HFmrEF/HFpEF phenotype and relatively better short-term outcomes.

Multiple interlinked pathophysiological mechanisms may be involved in the association of HGI with the HF phenotype and patient outcomes. First, a high HGI often means that the patient is producing excess AGEs at the same blood glucose level (15). *In vivo*, AGEs can bind to their receptor, RAGE, to induce oxidative stress and inflammatory cascade reactions, leading to abnormal cross-linking of collagen and elastin fibers in the extracellular matrix. These changes lead to arterial stiffness, myocardial fibrosis, and diastolic dysfunction, thereby promoting structural remodeling and functional deterioration in patients with HF (9, 16). This mechanism is particularly prominent in the pathogenesis of HFpEF (4, 5). Metabolic abnormalities such as diabetes mellitus are common in these patients, in whom the AGE/RAGE pathway is considered an important driving factor for myocardial vascular remodeling. The results of this study revealed that patients with a higher HGI tended to have the HFrEF phenotype, which might indicate that AGE-mediated myocardial injury might also accelerate the occurrence and development of HFrEF. Sustained hyperglycemia can directly damage cardiomyocytes and promote myocardial remodeling, aggravating the original cardiomyopathy or ischemic injury and leading to a decreased EF and the development of HFrEF. Mechanistically, the impact of HGI on cardiac remodeling may diverge across phenotypes. While AGE-mediated collagen cross-linking promotes stiffness typical of HFpEF, the ‘hyper-glycation’ state reflected by high HGI also exerts direct toxicity on cardiomyocytes. High levels of AGEs can trigger cardiomyocyte apoptosis and mitochondrial dysfunction via the RAGE-NF-κB pathway, leading to wall thinning and contractile failure (17). This cytotoxic effect may explain our finding that higher HGI is strongly associated with the HFrEF phenotype (pump failure) rather than just stiffness (18). Furthermore, regarding the risk of in-hospital decompensation, HGI serves as a marker for systemic vascular and renal burden. Elevated glycation can impair endothelial barrier function, increasing vascular permeability and susceptibility to pulmonary congestion. Concurrently, renal tubular glycation may induce diuretic resistance, necessitating the escalation of intravenous diuretic therapy observed in our high-HGI cohort (19).

Second, chronic inflammation and oxidative stress may be key factors through which the HGI is associated with the phenotype and prognosis of HF (20). A high HGI is often accompanied by long-term hyperglycemia, which not only promotes AGE accumulation but also triggers systemic inflammatory responses (21, 22). Inflammatory mediators can act on the cardiovascular system to cause myocardial apoptosis, fibrosis and vascular endothelial dysfunction, thereby aggravating cardiac insufficiency (23). Studies have suggested that inflammatory indicators such as the WBC count may play a partial mediating role between the HGI and adverse outcomes (24). Chronic low-grade inflammation may be more prevalent in patients with a high HGI, thereby contributing to phenotypes with lower LVEF and a higher incidence of In-hospital WHF. Notably, HFpEF is often regarded as an inflammation-driven HF subtype. Comorbidities such as obesity and diabetes mellitus can cause systemic inflammation and myocardial microvascular endothelial inflammation, ultimately leading to myocardial stiffness and impaired myocardial function (25). Therefore, the internal, high-glycation/high-inflammation environment reflected by the elevated HGI may contribute to the decline in cardiac function observed in patients with HFrEF and play a role in the pathogenesis of HFpEF.

The HGI essentially quantifies the difference in the degree of glycosylation of RBCs in an individual at a specific blood glucose level; this glycosylation is affected by many factors, such as genetic background, RBC lifespan, and enzyme activity (26–28). Patients with a high HGI may have a congenital or acquired predisposition to high glycation, which is reflected not only by high HbA1c levels but also by an increased susceptibility of tissues throughout the body to glycosylation damage (15). For instance, some studies have shown that among individuals with similar long-term average blood glucose levels, those with a higher HGI are more likely to develop diabetes complications and cardiovascular events (29, 30). Therefore, it can be speculated that HF patients with a high HGI experience more severe cumulative damage to myocardial and vascular tissues because of higher glycation sensitivity, resulting in more severe cardiac insufficiency and higher risks of. In contrast, an extremely low HGI may indicate another dangerous state: stress hyperglycemia even in the absence of a high degree of glycosylation, which is common in cases of a sudden increase in blood glucose under the stress of acute and severe diseases without an accompanying increase in HbA1c. This condition indicates a poor outcome in critical HF patients. However, among our study subjects (generally hospitalized patients), such patients accounted for a relatively small proportion. We mainly observed a relationship between elevated HGI and a poor outcome, which better aligns with the mechanism of chronic injury accumulation caused by a high HGI.

Notably, HFpEF and HFrEF patients differ significantly in metabolic characteristics. Previous epidemiological data have shown that approximately 40% of HF patients have diabetes mellitus (31). Metabolic syndrome-related obesity and insulin resistance are particularly common in HFpEF patients (32). Theoretically, these metabolic abnormalities can aggravate myocardial stiffness and diastolic function impairment through the AGE/RAGE-mediated pathway, thereby increasing the risk of progression to HFpEF. However, we observed that the HGI of patients in the HFpEF group was not higher than that of patients in the HFrEF group, which differs from what seems intuitive on the basis of the prevalence of diabetes mellitus. One possible explanation is that although HFpEF patients have a predisposition to developing hyperglycemia, the increase in HbA1c is limited by the shortened RBC survival period or other factors, so the glycation load reflected by the HGI is relatively underestimated. Notably, no studies in the literature can be compared with ours regarding our HF phenotypic stratification results. Our study fills this gap, suggesting that the HGI could serve as a new metric for understanding the differences in the metabolic backgrounds underlying different HF phenotypes. Consistent with recent evidence linking HGI to 1-year mortality (13, 14), our findings suggest HGI serves as a continuous risk marker. It reflects a cumulative glycemic burden that drives both acute in-hospital decompensation and chronic adverse outcomes, supporting its utility across the disease trajectory. In the future, more studies, especially those involving non-severe HF patients, are needed to verify our findings and to further explore the intrinsic differences between HFpEF and HFrEF in terms of glycation biology.

### Study limitations

This was a single-center retrospective study, and the associated selection bias and confounding residues may have resulted in a limited ability to draw causal inferences. All the participants were hospitalized HF patients without acute coronary syndrome or other acute diseases. Therefore, generalization of the results to other populations is limited. The HGI, which was calculated from FPG and HbA1c values obtained in a single measurement at admission, might be affected by stress status, RBC lifespan, and laboratory calibration differences and was not dynamically monitored. Additionally, mechanism indicators such as AGEs/RAGE binding were not determined. The biological causal chain that underlies our findings needs to be verified in prospective and mechanistic studies. Furthermore, the study endpoint was defined as therapy escalation (intensified diuretics or vasoactive agents) rather than hard endpoints like mortality. Although these events reflect significant hemodynamic instability, the lack of hard outcome data (such as post-discharge mortality or readmission) limits the interpretation of long-term prognosis.

Third, information regarding baseline Guideline-Directed Medical Therapy (GDMT), such as ARNIs, beta-blockers, and SGLT2 inhibitors, was not included in the multivariable adjustment due to incomplete documentation of pre-admission medication history in the electronic medical records. Since these therapies significantly impact cardiac remodeling and prognosis, their absence may introduce residual confounding. The observed associations between HGI and outcomes should be interpreted with this limitation in mind.

## Conclusion

In summary, our analysis demonstrated that an elevated HGI is independently associated with the HFrEF phenotype and the risk of in-hospital WHF. These findings suggest that HGI may hold value for risk stratification, though further prospective studies are needed to validate its clinical utility. In future studies, we will verify the threshold value of the HGI in a larger and more heterogeneous population, evaluate the incremental contribution of the HGI to clinical decision-making and outcome improvement, and explore how the HGI interacts with evidence-based treatments (such as SGLT2 inhibitors and ARNIs).

## Data Availability

The raw data supporting the conclusions of this article will be made available by the authors, without undue reservation.
